# Design and Optimization of Thermal Field for PVT Method 8-Inch SiC Crystal Growth

**DOI:** 10.3390/ma16020767

**Published:** 2023-01-12

**Authors:** Shengtao Zhang, Guoqing Fu, Hongda Cai, Junzhi Yang, Guofeng Fan, Yanyu Chen, Tie Li, Lili Zhao

**Affiliations:** 1 Harbin Institute of Technology, School of Chemistry and Chemical Engineering, Harbin 150001, China; 2 Harbin KY Semiconductor, Inc., Harbin 150028, China; 3 Soft-Impact China (Harbin), Ltd., Harbin 150028, China

**Keywords:** SiC, seed crystal cavity, growth interface, seed temperature

## Abstract

As a wide bandgap semiconductor material, silicon carbide has promising prospects for application. However, its commercial production size is currently 6 inches, and the difficulty in preparing larger single crystals increases exponentially with size increasing. Large-size single crystal growth is faced with the enormous problem of radial growth conditions deteriorating. Based on simulation tools, the physical field of 8-inch crystal growth is modeled and studied. By introducing the design of the seed cavity, the radial temperature difference in the seed crystal surface is reduced by 88% from 93 K of a basic scheme to 11 K, and the thermal field conditions with uniform radial temperature and moderate temperature gradient are obtained. Meanwhile, the effects of different processing conditions and relative positions of key structures on the surface temperature and axial temperature gradients of the seed crystals are analyzed in terms of new thermal field design, including induction power, frequency, diameter and height of coils, the distance between raw materials and the seed crystal. Meanwhiles, better process conditions and relative positions under experimental conditions are obtained. Based on the optimized conditions, the thermal field verification under seedless conditions is carried out, discovering that the single crystal deposition rate is 90% of that of polycrystalline deposition under the experimental conditions. Meanwhile, an 8-inch polycrystalline with 9.6 mm uniform deposition was successfully obtained after 120 h crystal growth, whose convexity is reduced from 13 mm to 6.4 mm compared with the original scheme. The results indicate that the optimized conditions can be used for single-crystal growth.

## 1. Introduction

Silicon carbide (SiC) materials are believed to have revolutionized the power electronics industry [1,2]. Its properties, such as a wide bandgap, high-temperature stability and high thermal conductivity, will bring SiC-based power devices a series of advantages [2,3,4]. In recent years, as new energy vehicle enterprises have employed SiC-based MOSFET modules for high-end vehicles, the application prospect of SiC substrate materials has again attracted extensive attention. SiC single crystal is prepared with the physical vapor transport (PVT) method, and 6-inch products are put into the market with thicknesses of about 10–30 mm. In contrast, after decades of development, monocrystalline silicon (Si), the cornerstone material of the modern electronic information industry, reaches the size of 12 inches. Meanwhile, its preparation method of the melt more easily contributes to crystal ingots with a greater thickness of up to more than 1 m, considerably reducing the substrate cost per unit area. Therefore, the current preparation method and the limitation of crystal size result in the steep market price of SiC substrates. Each substrate is sold at about USD 1000, severely limiting downstream industries [5] for further application.

Therefore, many analysts conduct research and development work on SiC crystal growth. The preparation of SiC crystals with the solution method has broad prospects but faces practical problems [6,7,8]. For example, because of the high melting point of SiC, adding a flux agent will cause inclusions and dislocations, and the continuous supply of carbon components remains unsolved. For the PVT method, increasing the thickness and size of the crystal is the key to reducing the preparation cost, which is also the main direction of researchers [5]. However, unlike the solution method, the gas phase material preparation is faced with lots of difficulties, such as the inability to observe directly, the vast change of raw material state, the deterioration of thermal field conditions during processing and the difficulty in regulating [9,10,11]. Therefore, it is not easy to increase the thickness and maintain high quality simultaneously and the potential of lifting space is limited in a short period of time [12]. SiC crystal growth has a natural habit of diameter expansion, so the expansion of crystal size has been the main direction in its industrialization in the past 30 years, from the initial crystalline grain to a centimeter-sized single crystal. Meanwhile, researchers and industry are moving from 5 cm, 7.5 cm, 10 cm and 15 cm to an 20 cm scale in recent years [13,14]. However, the enlargement of the crystal diameter is not a simple expansion by several times iterative growths. 

When the crystal size is enlarged, it is necessary to synchronously upgrade the thermal field conditions, maintain a certain axial temperature gradient, stabilize the driving force of crystal growth, and control the radial temperature uniformity of a large thermal field to secure a high crystal quality [15]. A SiC single crystal’s quality lies in minimizing macrodefects and microdefects, including polycrystalline, polytypes, micropipe, and dislocations. The control and optimization of process conditions is an important way to stabilize polytypes and reduce defect formation [16,17,18]. Research shows that SiC crystal growth is closely related to temperature and its gradient [19], where simulation methods can play an important role [20,21]. For a long time, the design of thermal field structures directly impacts crystal growth [22]. It is pointed out in the literature that the nearly flat and slightly convex morphology or profile is conducive to improving the quality of single crystals [13,23]. However, in the induction heating mode, the growth condition at the edge of the seed crystal is more likely to be different. The temperature gradient is too small because it is closer to the skin depth area of the crucible barrel, which may lead to a slow growth rate or even the decomposition of the seed crystal edge, severely restricting the growth of larger crystals.

To obtain the thermal field conditions and control methods for large-sized SiC crystal growth, the thermal field of crystal growth was modeled by simulation tools. Under the initial thermal field conditions, the temperature suitable for seed crystal growth is obtained. Still, the radial temperature difference of the seed reaches 93 K. First, by introducing the novel design of the seed cavity, the thermal field conditions of a radial temperature difference of about 10 K and an axial temperature gradient of 12 K/cm are observed. Then, the influence of several key factors on the seed crystal interface temperature and temperature gradient distribution is analyzed, including induction coils frequency and power, the height of raw materials and coils, and the diameter of coils, from which better parameter ranges are obtained. Based on the optimized thermal field conditions, the thermal field verification is carried out, and a homogeneous polycrystalline deposition with a thickness of 9.6 mm is successfully prepared. The experiment shows that the convexity of the optimized scheme is 3.4 mm less than that of the original scheme without a seed crystal, as the standard deviation of thickness distribution is reduced from 5.4 to 2.6. The experiment also verifies that the growth rates of a single crystal (using small wafers bonded on graphite plates) and a polycrystal (using graphite plates) are comparable under the experimental conditions of this paper for crystal growth. This work provides essential references for optimizing the thermal field of large-size crystal growth, both in regulating SiC seed crystal deposition conditions and the design concept of the thermal field.

## 2. Modeling and Experiment

**Figure 1:**
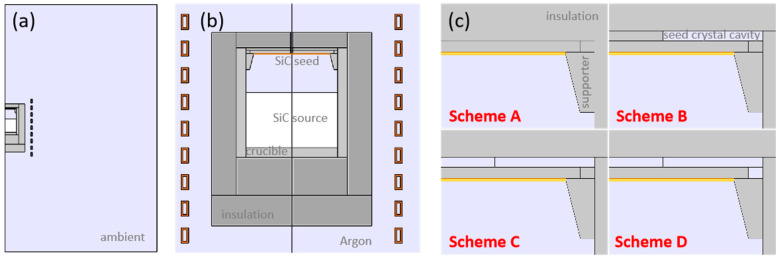
Thermal field structure and schemes. (**a**) Whole computational domain; (**b**) The physicalfield; (**c**) Thermal field details under different schemes.

The induction power supply provides the energy required for crystal growth, which has the advantage of high efficiency. An alternating current forms the alternating magnetic field. Then, the induced current is formed in the side wall of the crucible, which is used as the heat source to provide the temperature conditions required for the growth of 8-inch crystals. Previous studies have shown that the near-flat and microconvex temperature distribution and uniform temperature gradient are crucial for high-quality crystals, significantly when the crystal size is expanded to 6 inches and above. Given the above requirements, focusing on the research of the temperature distribution of the thermal field, the main structures and materials of the equipment and thermal field are abstracted, and its profile is shown in Figure 1. Different materials are marked with different colors. Since a quartz tube instead of a water-cooled counterpart is used as a furnace wall, supporting the bottom of the thermal field, most of the heat is directly transferred to the environment. In contrast, the furnace wall and support structures are ignored (Figure 1a). The thermal field is located in the center of the induction coils. In the default position, the middle of the coils in the thermal field is 95 mm lower than the raw material surface. The thermal area consists of a thermal insulation layer, graphite crucible and internal structure from the outside to the inside, SiC seed crystal, seed crystal supporter, gas chamber and SiC raw materials from top to bottom (Figure 1b). The gas pressure is set at 1000 Pa. A temperature-measuring hole with a diameter of 6 mm is reserved in the center of the top insulation layer. The thermometer (Endurance™ 1RH) used in the experiment is manufactured by Fluke Process Instruments, whose repeatability is ±0.3% full scale and system accuracy is ±0.5%T_meas_. The parameters of the main structure of the model are shown in Table 1, where the diameter and height of the main structures are marked.

To solve the problem of the vast temperature difference between the edge and center of the seed crystal, various thermal field structures are designed to compare and study their effects on the temperature distribution of the seed crystal (Figure 1c). In the initial scheme A, the upper part of the seed crystal is the crucible upper lid or seed crystal holder as the insulation layer, and the side of the seed crystal is the support structure. Scheme B integrates the design of the seed crystal cavity into the structure, so there is a gas chamber with a thickness of 7 mm at the top of the seed crystal with a diameter range from 100 mm to 240 mm. Scheme C directly extends the gas chamber above the whole seed crystal. In addition to the gas chamber at the top, Scheme D also provides a small chamber between the seed crystal holder and the inner wall of the crucible on the side of the seed crystal connecting with the top gas chamber. Other structures remain the same under the four designs.

Table 2 shows the physical parameters of the primary materials, including graphite, felt, crucible graphite, SiC material and gas. The gas in the model is set as argon for calculation simplification. It should be pointed out that the actual preparation of conductive SiC single crystal demands mixing 5–20% nitrogen with argon to provide the carriers required by the materials. Among them, the heat transfer coefficient of the material changes nonlinearly with temperature. During the calculation, the power needed for the simulation and experiment is appropriate by adjusting the thermal conductivity of the insulation layer. The details description of the correlation function can be found in the Supplementary Electronic Materials.

In establishing the model, the steady state calculation of the electromagnetic heat is based on the input power of the coils. The problem of electromagnetic analysis on a macroscopic level lies in solving Maxwell’s equations subject to certain boundary conditions. The shifting current forms an alternating magnetic field, which generates the induced current and heat. The electromagnetic heating node represents the electromagnetic losses, *Qe* (W/m^3^), as a heat source in its transfer part of the model. It is given by:

(1) $$\rho C_{P}u\cdot \nabla T=\nabla \cdot \left(k\nabla T\right)+Q_{e}$$

(2) $$Q_{e}=Q_{vh}+Q_{ml}$$

(3) $$Q_{rh}=\frac{1}{2}Re\left(J\cdot E^{*}\right)$$

(4) $$Q_{ml}=\frac{1}{2}Re\left(i\omega B\cdot H^{*}\right)$$

*Q_rh_* are the resistive losses, and *Q_ml_* are the magnetic losses. The Frequency-Stationary calculation is adopted in this calculation. Meanwhile, there are many introductions on the basic principles of heat transfer in the simulation process in the published literature [24,25,26]. The influence of the magnetic field on solids and fluids in three-dimensional structures has also been introduced [27,28]. Under the temperature of thousands of degrees, radiation heat transfer is a nonnegligible process. We introduce surface-to-surface radiation, and the hemi-cube method is selected. The emissivity of the material surface comes from the definition of the physical parameters of the material itself.

**Figure 2:**
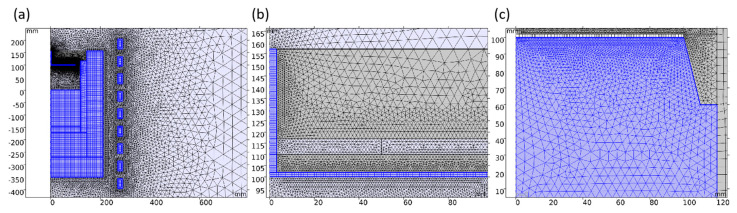
Generated grids. (**a**) Thermal field; (**b**) seed crystal and view tube; (**c**) growth chamber.

In the mesh generation procedure, the grids are manually set and optimized. Generally speaking, the hexahedral mesh has the advantages of great accuracy and efficiency, while the tetrahedral mesh is more suitable for dealing with complex structures. Various strategies are adopted to secure better grid quality and computing speed, considering the different shapes of thermal field domains. Table 3 shows the grids’ setting details, and Figure 2 shows the grids’ diagram after setting. The hexagonal structure grids are adopted for temperature measuring windows, seed crystal, crucible side wall and raw materials, whose grids cell size is set to be much smaller than its geometric size. According to the assessment, the mesh elements’ quality reaches 0.9966 on average. The total quality of the generated grids is above 0.9, indicating that the grid settings are highly qualified.

Since the research mainly focuses on the temperature distribution of crystal growth interface, the data consistency of power and temperature is the key to judge the fitting degree between simulation and actual data. On the one hand, the thermal insulation layer directly affects heat transfer. On the other hand, the consistency of the natural thermal insulation materials batches is not very good. Even the matching and use of details result in the actual physical parameters deviating from the nominal value. Therefore, the fitting progress with the experiment is mainly carried out by adjusting the heat transfer coefficient of the thermal insulation layer. Based on the initial thermal field scheme, after multiple adjustments, the difference between the temperature of the simulated temperature measuring point and the measuring value is within 10 K under the same induction power, which is close to the accuracy of the thermometer used. It can be considered that the thermal field simulation meets the requirements. Details for the boundary conditions are shown in Table 4. By default, the induction coil power is 13 kW, and the frequency is 14 kHz, which will be adjusted later according to the conditions studied. 

## 3. Results and Discussion

### 3.1. Optimization of Seed Crystal Interface Temperature

#### 3.1.1. Temperature Distribution Inside Crucible

**Figure 3:**
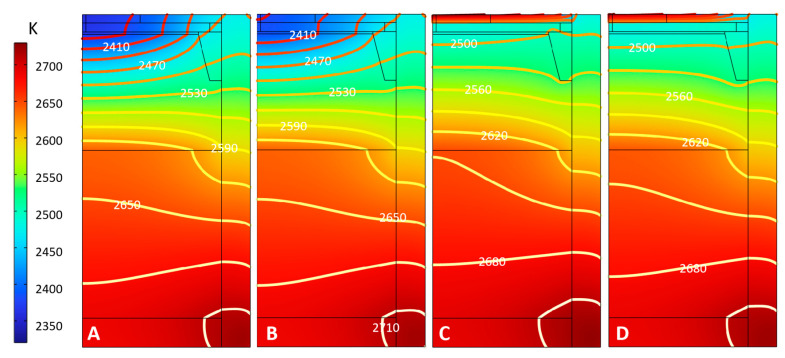
Temperature distribution in the crucible. (**A**) Scheme A; (**B**) Scheme B; (**C**) Scheme C; (**D**) Scheme D.

The internal temperature distribution of the crucible under different schemes is shown in Figure 3. The temperature distribution characteristic inside the crucible is similar, as the high-low temperature zones are located at the crucible’s lower and upper parts. The temperature reaches 2700 K in the high-temperature zone, while the value in the low-temperature zone does not exceed 2450 K. The internal temperature of the raw materials is mostly between 2650 K and 2750 K. Due to the small heat transfer efficiency of the cavity above the SiC raw materials, the temperature of the raw materials surface is around 2600 K, forming a temperature difference of about 200 K with that of the seed crystal near 2400 K. Through calculation, the temperature gradient in the crucible growth chamber reaches 10–20 K/cm, and the seed crystal temperature is around 2400–2500 K, which is close to the public reports. This shows that the temperature and gradient at the seed obtained under the basic thermal field design scheme can meet the SiC single crystal growth requirements. However, due to the influence of the change of top insulation materials, the temperature distribution at the seed crystal under different schemes varies greatly. The existence of temperature-measuring holes at the top under Scheme A and Scheme B makes the seed crystal temperature significantly lower.

**Figure 4:**
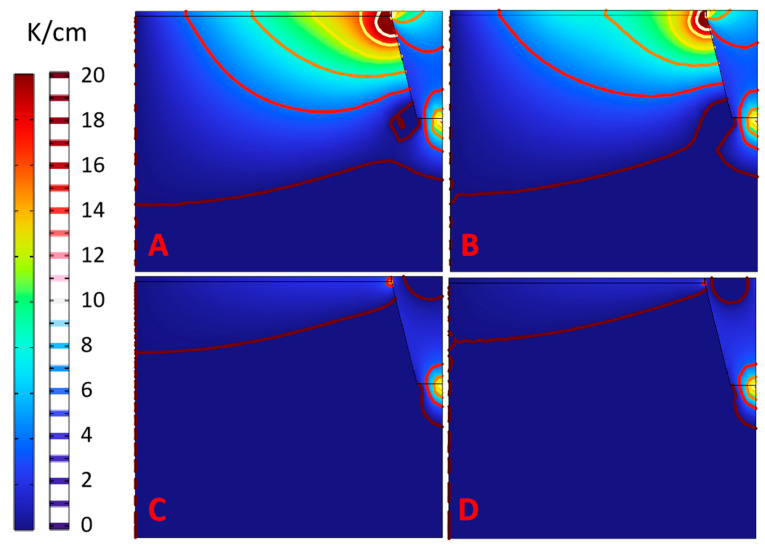
Radial temperature gradient in the chamber. (**A**) Scheme A; (**B**) Scheme B; (**C**) Scheme C; (**D**) Scheme D.

The radial temperature gradient distribution better illustrates the temperature distribution characteristics more obviously, as shown in Figure 4. Scheme A has the largest radial temperature gradient of the seed crystal. There are more than 20 isotherms in the radius of the seed crystal, and the edge temperature gradient reaches 20 K/cm. As a result, the temperature difference between the center and the edge of the seed crystal is remarkable, and this scheme is not suitable for uniform single-crystal growth. Compared with Scheme A, Scheme B has fewer isotherms and a smaller radial temperature gradient, indicating that the top seed cavity plays a positive role. The radial temperature gradient under Scheme C and Scheme D presents completely different characteristics. Values in the seed crystal and even the whole growth chamber are below 2–4 K/cm. Meanwhile, the temperature gradients at the interfaces between the supporter and the seed crystal, the supporter and crucible, crucible side wall and supporter are great. Compared with Scheme C, Scheme D maintains a smaller radial temperature gradient at the edge of the seed crystal and has a much better radial temperature gradient distribution.

#### 3.1.2. Temperature Distribution in SiC Seeds

**Figure 5:**
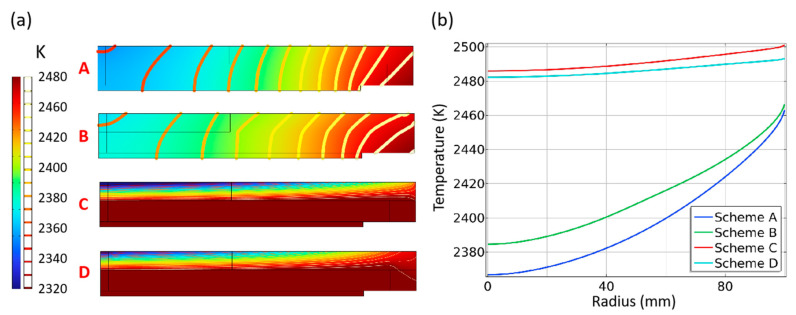
(**a**) Temperature distribution of the seed crystal (**A**) Scheme A; (**B**) Scheme B; (**C**) Scheme C; (**D**) Scheme D; (**b**) seed crystal surface temperature of different schemes.

The temperature distribution of the seed crystal, the seed crystal holder, and the temperature distribution of the seed crystal interface clearly show the differences between the four schemes, as shown in Figure 5. The adjacent isotherms are set with every 10 K temperature difference. Due to the existence of the seed crystal cavity, the isotherms of Scheme C and D are greatly compressed into the top of the seed crystal holder, which has a better thermal insulation effect and hinders heat loss, while only one isotherm appears in the seed crystal holder (Figure 5a). The isotherm inside the seed holder of Scheme D is closer to the edge than that of Scheme C, which means the edge temperature is reduced, which is affected by the seed cavity on the side of the seed holder. Figure 5b shows the temperature distribution of the seed crystal under different schemes. The average temperature of the seed crystal surface under the four schemes is 2415 K, 2425 K, 2493 K and 2488 K, respectively, and the temperature difference of the seed crystal surface is 93 K, 82 K, 16 K and 11 K separately. Compared with the original scheme, the radial temperature difference of the improved scheme is reduced by 88%. Consistent with the foregoing analysis, the existence of the seed crystal cavity directly affects the temperature distribution on the seed crystal surface by changing the heat transfer on the back and side of the seed crystal, greatly improving the radial temperature consistency.

### 3.2. Influence of Induction Coils Characteristics

#### 3.2.1. Heating Frequency

**Figure 6:**
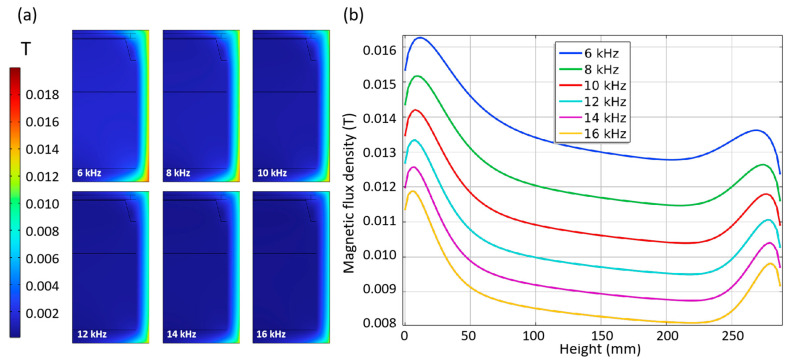
(**a**) Magnetic flux density (MFD) of Crucible; (**b**) MFD along the outer wall of crucible.

Due to the skin effect, under the condition of induction heating, heat is always generated at a depth of several millimeters near the surface of the heating body and then transferred to the inside of the thermal field. As shown in Figure 6, the magnetic flux mode distribution in the crucible is mainly concentrated on the side wall of the crucible, especially at the top and bottom. With heating frequency (f) increasing, the magnetic flux on the crucible surface decreases gradually, with average values shifting from 0.0135 T at 6 kHz to 0.009 T at 16 kHz. Figure 6b shows the magnetic flux curve along the side wall of the crucible, where a height of 0 mm corresponds to the bottom of the side wall of the crucible. As the coil center is closer to the bottom of the crucible, the magnetic flux at the bottom of the crucible is significantly greater than that in other areas, and the maximum parameter is about 30% higher than the minimum. The change rate of magnetic flux determines the magnitude of the induced current, which will significantly impact the temperature distribution.

**Figure 7:**
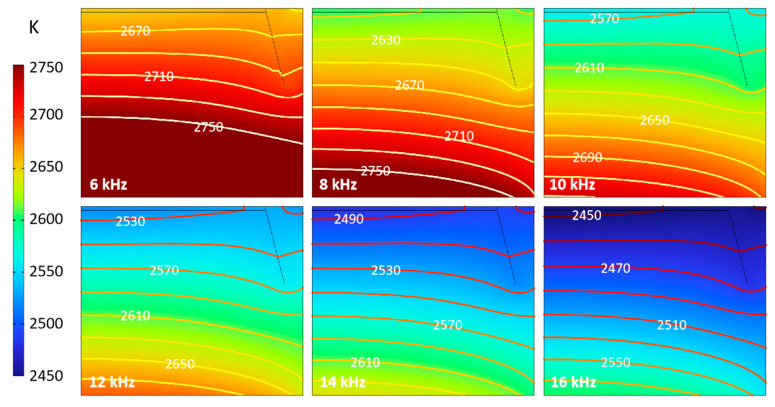
Temperature distribution in chamber.

Penetration depth δ in the skin effect is proportional to f^−1/2^, and changing the heating frequency is equivalent to directly changing the effective thickness of the heater, thus indirectly affecting the heat transfer process. The temperature distribution in the chamber under different frequencies is shown in Figure 7. With the frequency increasing, the temperature in the reaction chamber falls gradually due to the decrease in penetration depth. When the frequency increases by 2 kHz, the temperature in the corresponding chamber decreases by about 40 K, but the temperature difference in the chamber always reaches above 150 K. It is found from the isotherms that the distribution of isotherms in the chamber at different frequencies is very similar. The isotherms near the seed crystal show a downward, nearly flat and slightly convex shape. Meanwhile, the area near the middle part of the support shows a downward, slightly convex shape, especially when it is far away from the seed crystal. This indicates the crystal tends to form an M-shaped unfavorable shape when the crystal grows thicker. It is necessary to further analyze and compare the effects of changes in crystal thickness.

**Figure 8:**
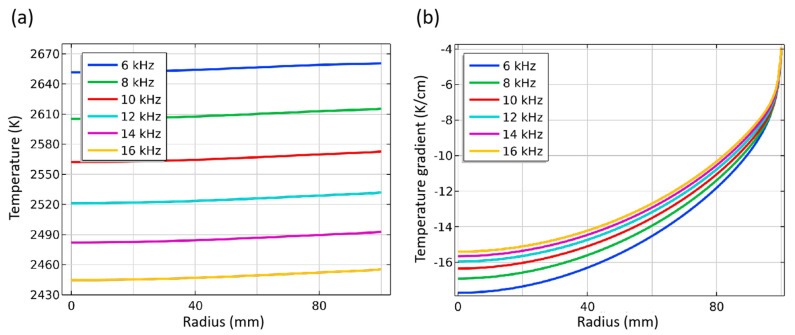
Temperature and temperature gradient at different frequency. (**a**) Temperature along surface of seed crystal; (**b**) Temperature gradient around seed crystal.

The temperature and temperature gradient distribution curve on the seed crystal surface can help better analyze the growth conditions, as shown in Figure 8. The temperature of the seed crystal interface decreases with the increase of frequency of about 50 K per 2 kHz. No matter the frequency, the temperature at the edge of the seed crystal is slightly higher, about 10 K than that of the center part (Figure 8a). The temperature gradient around the seed crystal surface in most areas is above −8 K/cm. A larger value characterizes it in the center but a smaller value in the edge, which is consistent with the required slight convex crystal growth interface morphology. With the increase in frequency, the temperature gradient decreases, and the gradient in the middle of the seed crystal decreases more than that in the edge (Figure 8a). This shows that the uniformity of the temperature gradient of the seed crystal surface can be improved by increasing the frequency, but the temperature of the seed crystal surface can be reduced simultaneously.

#### 3.2.2. Heating Power

**Figure 9:**
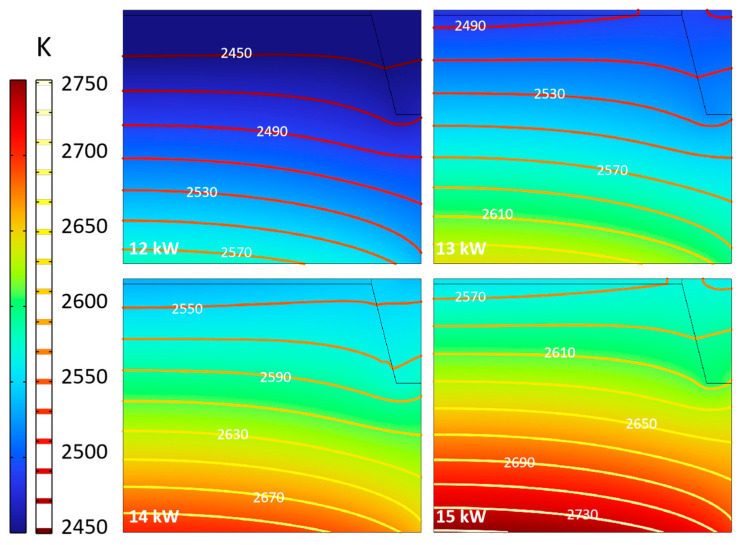
Temperature distribution in the chamber.

The heating power is the source of the temperature conditions during the crystal growth process. With the increase in power, the temperature inside the thermal field is significantly increased, as shown in Figure 9. When the power is increased by 1 kW, the temperature in the chamber is increased by about 40–60 K, which is well matched with the temperature change of about 5 K for every 0.1 kW in the actual experimental, indicating the reliability of the model. In the growth chamber, the isotherms’ density increases with the increase of power. The temperature difference inside the chamber increases from about 140 K at 12 kW to 160 K at 14 kW. Therefore, the temperature difference inside the chamber increases by about 10 K every 1 kW, indicating that the temperature gradient also rises. Another phenomenon is the shape of the isotherm. The curve is smoother when the power is lower, indicating that the radial consistency of the seed crystal surface is good in this situation. The conclusion is consistent with the above frequency analysis. Under the same conditions, increasing the heating power or penetration depth is conducive to improving the temperature inside the crucible and therefore reducing the radial temperature consistency.

**Figure 10:**
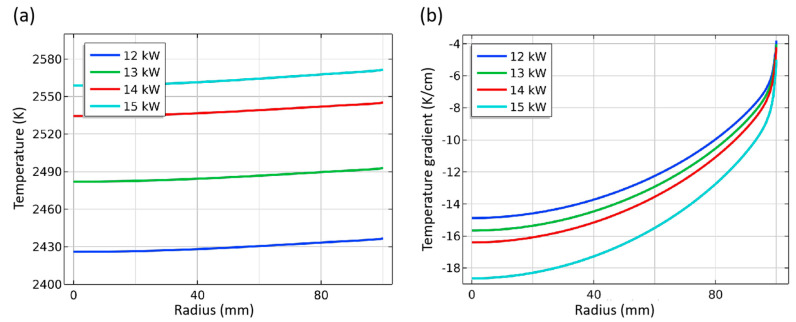
Temperature and temperature gradient at different power. (**a**) Temperature along surface of seed crystal; (**b**) Temperature gradient around seed crystal.

The effects of increasing power on the temperature and temperature gradient at the seed crystal interface are shown in Figure 10. With the power rising, the temperature of the seed crystal interface increases by about 60 K, 40 K, and 12 K, respectively. It reveals that the effect of increasing the temperature of the seed crystal interface decreases gradually. This means the whole thermal field has formed a new heat transfer equilibrium state under different power. When the high-temperature zone reaches a higher temperature due to the enhanced heat transfer effect, the temperature at the seed crystal is affected by the cold point of the top temperature measuring window, and its increase is not as obvious as the overall shift in the chamber. The change in temperature gradient is consistent with the above analysis. With the power rising, the temperature gradient increases greatly, and the central part of the seed crystal increases even more than the edge, which increases the difference in radial deposition conditions. This shows that high power is conducive to obtaining a large temperature gradient without changing the thermal field structure but at the expense of deposition uniformity; therefore, it needs careful consideration.

### 3.3. Relative Position of Coil and Raw Material

#### 3.3.1. Diameter of Induction Coils

**Figure 11:**
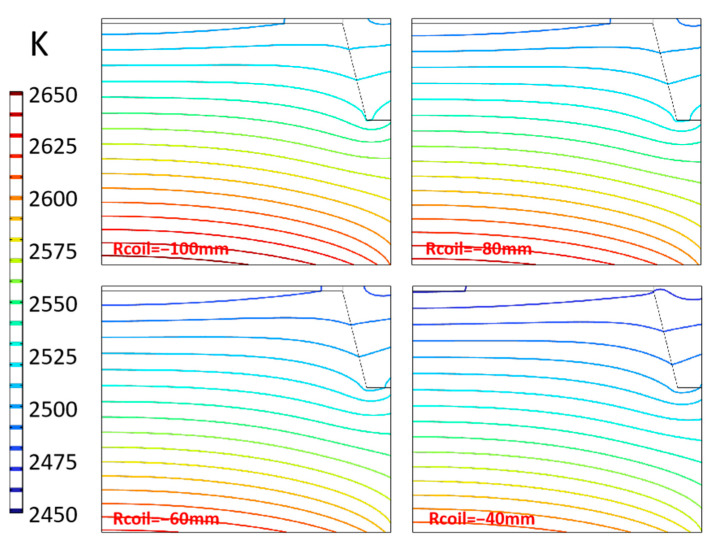
Isotherms distribution in the crucible.

According to the principle of induction heating, the characteristics of the induction coils and the position of the heating body directly determine the distribution characteristics of the inductive magnetic flux. The radius of coils is represented by “Rcoil”. The default value of the coils’ diameter is marked as −80 mm, and −100 mm means that the coils’ diameter is 40 mm smaller than the default value. The temperature distribution in the chamber with different coils diameters is shown in Figure 11. With the coils’ diameter increasing, the temperature inside the chamber decreases gradually, but the shape and density of the isotherms do not change significantly. This is because the change in the coil’s diameter directly affects the distance between the coils and the induction heating body, thus changing the magnetic induction line density in the induction area. However, compared with the diameter of coils that exceed 400 mm, the diameter change of 40 mm range has little effect. Therefore, it can be approximately considered that the heating efficiency has changed, and there is no change in the position of the relatively high-temperature area and the relatively low-temperature area. As the change of coils’ diameter is much smaller than that of coils and heaters, the effect on temperature is very limited. It should be pointed out that the adjustment of this distance will involve different heating efficiency and the limitation on equipment size, which should be considered.

**Figure 12:**
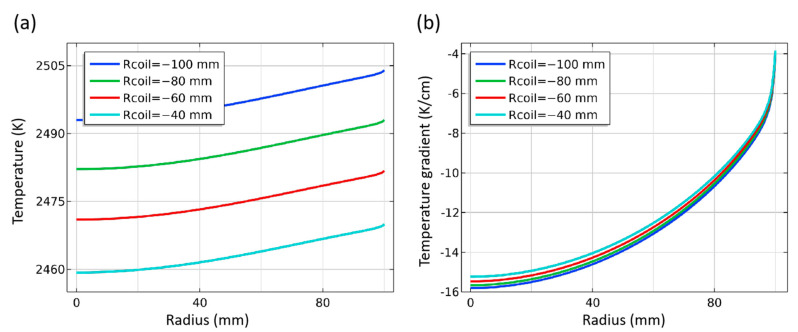
Temperature and temperature gradient at different coils diameter. (**a**) Temperature along surface of seed crystal; (**b**) Temperature gradient around seed crystal.

The influence of coil diameter on the deposition conditions at the seed crystal is shown in Figure 12. Under the experimental conditions, the temperature at the seed crystal interface decreases by about 11 K with the increase of the coils’ diameter by every 40 mm, and the decrease range increases. The temperature gradient decreases slightly with the coils moving away from the thermal field. Still, the total amplitude is within 1 K. This shows that when the relative distance between the coils and the heater is within a reasonable range, the influence on the temperature gradient can be ignored. Still, it will directly affect the temperature of the seed crystal interface. A smaller space between the coils and crucible is recommended to improve heating efficiency and save electric energy. However, in practice, the coil needs to be embedded with circulating water for cooling, and there are two equipment structures distinguished by position relative to the furnace shell: the exterior coils type and the built-in coils type. Coils in the former equipment are often separated from the thermal field by quartz structure, and there is a certain distance between the thermal field and the quartz tube, as well as the quartz tube and the thermal field. Coils can be closer to the former equipment’s thermal field, but the short circuit problem caused by too close proximity should be avoided. At the same time, the thermal insulation layer will also be affected by the alternating magnetic field of induction heating to produce induced current, which is closer to the coils than the crucible. It is about two orders of magnitude lower than the crucible heating body, according to the calculation, which also needs to be taken into account.

#### 3.3.2. Position of Induction Coils

**Figure 13:**
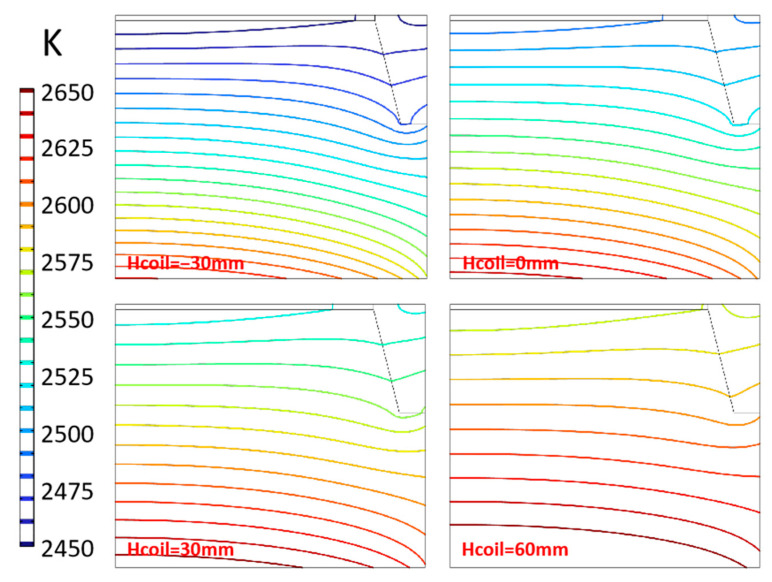
Isotherms distribution in chamber.

The height of coils directly affects the position of the high-temperature zone and significantly affects the temperature and temperature gradient distribution. The coil height is represented by “Hcoil” in the simulation. By default, the coil height is marked as 0 mm, and −30 mm means that the coils are moved down 30 mm from the default position. The temperature distribution in the chamber at different coil positions is shown in Figure 13, and the temperature difference between adjacent isotherms is 10 K. With the increasing of the coils’ position, the temperature in the chamber increases gradually, and the isotherms distribution becomes sparse. The temperature difference in the chamber decreases from about 200 K to 110 K, which means that the temperature difference in the chamber decreases by 30 K for every 30 mm drop, reducing the temperature gradient in the chamber. The shape of the isotherm differs slightly at different positions. When Hcoil = −30 mm, the isotherms’ distribution near the seed crystal is slightly convex, while when the parameter is 60 mm, the isotherms become smoother, which will adversely affect the consistency of the temperature gradient.

**Figure 14:**
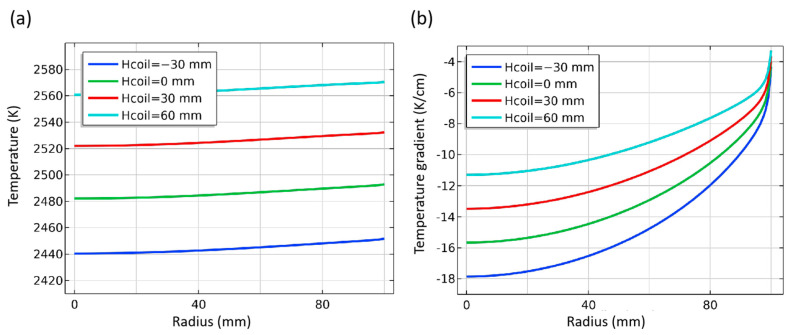
Temperature and temperature gradient at different coils height. (**a**) Temperature along surface of seed crystal; (**b**) Temperature gradient around seed crystal.

As shown in Figure 14, the higher the position of the coils, the higher the temperature area is closer to the seed crystal surface. The temperature rises about 40 K, 38 K, and 36 K, respectively, for every 30 mm increase in coil position. That means that with the coil position increasing, the effect of increasing the seed crystal surface temperature decreases (Figure 14a). The temperature gradient distribution changes significantly, and the value in the middle of the seed crystal decreases by about 2.7 K/cm for every 30 mm rising. The height change of 90 mm from −30 mm to 60 mm makes the temperature gradient in the middle of the seed crystal decrease by nearly half. At the same time, the value at the edge of the seed crystal also changed significantly, but the range of the radial temperature gradient decreased apparently. This indicates that within the range of parameters set in the experiment, increasing the coils’ position is conducive to obtaining higher crystal surface temperature and a more uniform radial temperature gradient, but at the expense of axial temperature gradient. In conclusion, when adjusting growth conditions by the relative height of the coils or thermal field, it is necessary to balance the consistency of temperature gradient and temperature, which can be coordinated with heating power and frequency for best results.

#### 3.3.3. Position of SiC Source Surface

**Figure 15:**
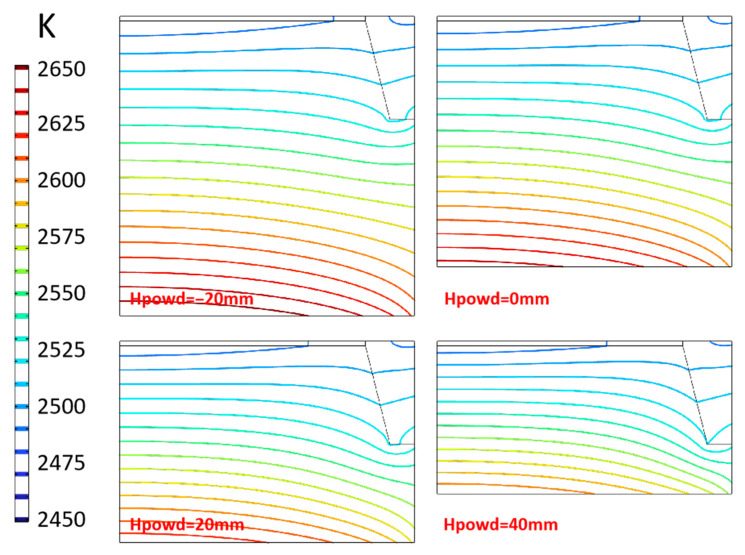
Isotherms distribution in chamber.

As the main solid material in the crucible, raw materials, the gas area, and the seed crystal are in the growth chamber. The height of the raw material directly changes the distance between the seed crystal and the raw material, which will have a certain impact on the temperature distribution. The position of the raw material surface is expressed as “Hpowd”. By default, the height of the raw material is marked as 0 mm, and −20 mm means that the raw material surface is 20 mm lower than the default position. The temperature distribution in the chamber under different heights of raw materials is shown in Figure 15. With the position of the raw material surface rising, the space of the chamber is compressed. The temperature at the bottom of the growth chamber, that is, the raw material surface also decreases. This is because under the conditions that radiation heat transfer is dominant, the raw material has a porous powder structure, and its height directly affects the thermal resistance of this area, thus affecting the heat transfer process. At the same time, due to the change in the distance between the raw material level and the seed crystal, the temperature of the seed crystal surface rises due to the dominant influence of the distance on the radiative heat transfer.

**Figure 16:**
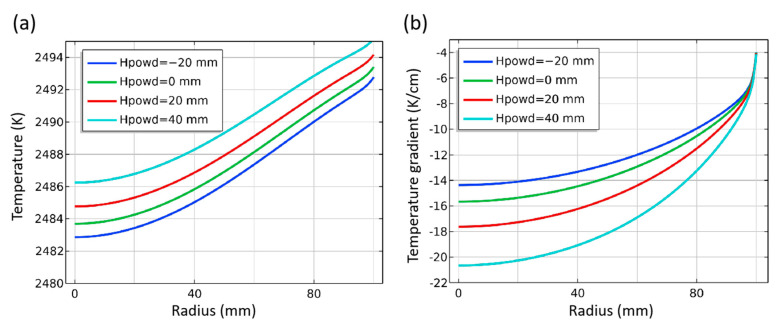
Temperature and temperature gradient at different powder height. (**a**) Temperature along surface of seed crystal; (**b**) Temperature gradient around seed crystal.

With the raw material surface’s position increasing, the seed crystal’s surface temperature gradually increases simultaneously, and the increase in scope becomes larger (Figure 16a). From −20 mm to 40 mm, the surface temperature of the seed crystal center increases by about 1 K, 1.2 K and 1.4 K, respectively, for every 20 mm increase in the raw material surface. It can be seen that the increasing amplitude is not obvious, indicating that the effect of raw materials on the heat transfer of the crucible wall is similar to that of reducing the heat transfer distance between raw materials and seed crystals or increasing the radiation heat transfer. However, the axial temperature gradient distribution on the seed crystal surface changes more markedly (Figure 16b). With the raw material surface increasing, the temperature gradient increases by 1.2 K/cm, 2 K/cm, and 3 K/cm, respectively. When the raw material surface reaches a height of 40 mm, the value difference between the seed crystal center and the position with a radius of 80 mm reaches 6.5 K/cm. This shows that within the range of the parameters set in the experiment, increasing the height of the raw material surface or cutting down the distance between raw materials and the seed crystal is profitable to increase the surface temperature and temperature gradient of the seed crystal, thereby increasing the crystal growth rate, but count against building a radial uniform temperature gradient.

**Figure 17:**
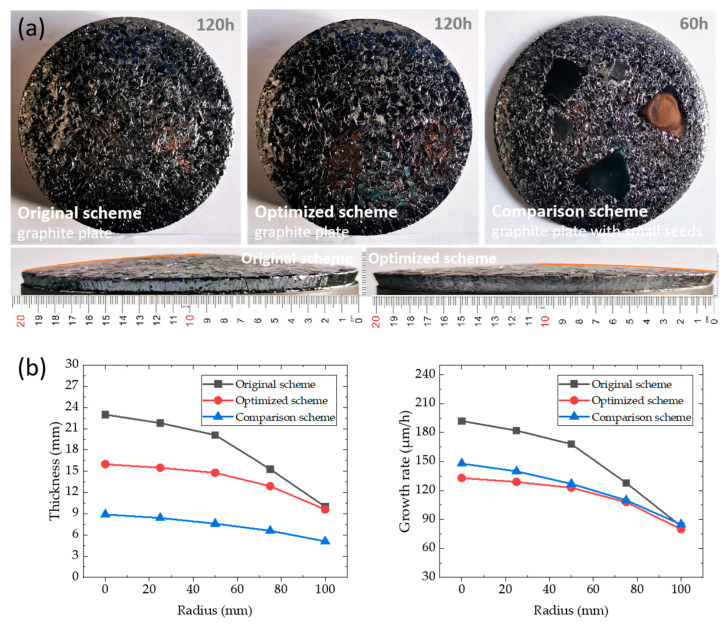
(**a**) 200 mm polycrystalline; (**b**) crystal thickness and growth rate.

Previous literature points out that the polycrystals deposition rate under seedless crystal is comparable with a single crystal, indicating that the single crystal and polycrystals always have a similar profile. Polycrystal deposition growth is actually a process of grain growth after multi-point nucleation. Compared with single crystal growth with the seed crystal, its process is not strictly limited by the step-flow mode and single crystal orientation. To verify the simulation results, a graphite plate was used to replace the seed crystal for crystal growth experiments. The results of the actual crystal and its thickness are shown in Figure 17. Three groups of experiments include the graphite plate as the seed crystal under the original scheme, the graphite plate as the seed crystal under the optimized conditions, and the graphite plate bonded with small crystal chips as the seed crystal under the optimized conditions (the comparison scheme). The crystal growth processes are carried out for 120 h, 120 h and 60 h, respectively. In the original scheme, the crystal is more convex, and the grain size at the center is different from that near the circumference. The maximum crystal thickness under this condition is 23 mm (growth rate is 192 μm/h), and the minimum thickness is 10 mm (growth rate is 83 μm/h), whose convexity is 13 mm. Under the optimized scheme, the maximum crystal thickness is 16 mm, the minimum thickness is 9.6 mm, and its convexity is 6.4 mm. Under the comparison scheme, the crystal surface is relatively flat, similar to the optimized scheme, and the bonded small crystal chips have a relatively uniform deposition. The polycrystalline grains in the comparison scheme are smaller because the fusion of small grains occurs with the progress of crystal growth. Under the original scheme, the maximum radial growth rate of the crystal is 2.3 times the minimum growth rate, while the value is 1.7 under the optimized scheme. The growth rate distribution of the latter is around 100–130 μm/h, and its growth rate uniformity is greatly improved. Under the optimized scheme, the average value of the crystal growth rate is slower because the axial temperature gradient decreases after introducing the seed cavity as the driving force of crystal growth. For graphite plates bonded with small wafers, the thickness of the single crystal area and polycrystalline area on the same circumference is closed. After deducting 0.8 mm of wafer thickness, the growth rate of a single crystal is about 90% of the growth rate of the surrounding polycrystalline. Polycrystals with a diameter of 200 mm, a thickness of about 10 mm and a nearly flat and slightly convex profile were successfully prepared by optimizing the conditions. Therefore, the optimized scheme can provide better crystal growth conditions in terms of deposition uniformity. 

Meanwhile, the experiments verified that under the conditions of a seedless crystal, polycrystal growth could be carried out using alternative materials, such as a graphite plate, as the exploration method for single-crystal growth.

## 4. Conclusions

Based on the electromagnetic heating, heat transfer and surface radiation modules of simulation software, the thermal field model of an 8-inch SiC crystal growth of induction heating PVT equipment was established for the first time. After fitting and adjusting with the experiment, the temperature difference between the simulation and experiment was close to 10 K, showing the reliability of the simulation results. Aiming at the problem of poor radial homogeneity of temperature conditions in the growth of large-size crystals, the seed crystal cavity is proposed for the first time to improve the local temperature distribution. The study shows that the temperature difference on the surface of the seed crystal in the new design was reduced by about 88% from 93 K to 11 K, and the temperature conditions suitable for crystal growth were successfully obtained. 

Meanwhile, the characteristics of the induction coils and the positions of several key structures are also studied and analyzed. First, higher frequency and lower power are conducive to improving the uniformity of the seed surface temperature gradient but will reduce the surface temperature. Second, a smaller coil diameter is conducive to improving the surface temperature of the seed crystal and heating efficiency while having little effect on the temperature gradient. Third, increasing the relative height of the coils is conducive to improving the uniformity of the temperature gradient, but it will reduce the temperature gradient value. Fourth, a closer distance between the raw material surface and the seed crystal is conducive to increasing the temperature and temperature gradient. Still, it aggravates the non-uniformity of the radial conditions. 

Finally, three experiments were conducted to verify the simulation results. The uniformly deposited 8-inch polycrystals with 9.6 mm thickness and 110 μm/h average growth rate are successfully prepared under the optimized conditions, and the convexity of the crystal is reduced by 50% compared with the original scheme, which verifies that the optimized schemes can provide conditions for the growth of large single crystals. In addition, the results show that polycrystalline has similar growth rates with single crystal under the same conditions, supporting the feasibility of thermal field conditions can be verified by polycrystalline deposition for the PVT method.

## Figures and Tables

**Table 1:** Parameters of main structures.

Structure	Diameter (mm)	Height (mm)
Ambient	3200	2600
Insulation	420	507
Crucible	290	287
SiC seed	200	2
SiC source	200	145
Coils	40 × 20 mm (with cavity 30 × 10 mm)	

**Table 2:** Physical Parameters of Main Materials.

Parameter	Unit	Graphite Felt	Graphite	SiC	Argon [gas]
Conductivity	S/m	200	sigGR(T)	1000	0
Heat capacity (P)	J/(kg·K)	1000	2260	1200	C_gas_2(T)
Density	kg/m^3^	100	1950	3200	rho_gas_3(T)
Thermal conductivity	W/(m·K)	kGF(T)	kGR(T)	kSiC(T)	k_gas_3(T)

**Table 3:** Control parameters and quality of the generated mesh.

Domains	Mesh Type	Maximum Cell Size (mm)	Grid Cell Number	Mesh Element Quality
Gas domain	Triangular	44	25,926	0.8968
Gas inside crucible	Triangular	10		
Coils	Quadrilateral	5	4144	0.9966
Side and bottom insulation	Quadrilateral	5		
Crucible wall, bottom and powder	Quadrilateral	5		
SiC seed and view tube	Quadrilateral	1		

**Table 4:** Boundary conditions.

Parameter	Unit	Structure	Value
Temperature	K	Surface of coils	300
		Ambient	300
Pressure	Pa	Fluid domains	1000
Default power	kW	Coils	13
Default frequency	kHz	Coils	14

## Data Availability

Data are contained within the article.

## Supplementary Materials

The following supporting information can be downloaded at: https://www.mdpi.com/article/10.3390/ma16020767/s1, The supporting materials is a supplementary description of some parameters in Table 2.
