# Supplementary figures and images for: Design and Optimization of Thermal Field for PVT Method 8-Inch SiC Crystal Growth
Source: Materials (Basel). 2023 Jan 12;16(2):767. doi: 10.3390/ma16020767 (PMC9867089; doi:10.3390/ma16020767)

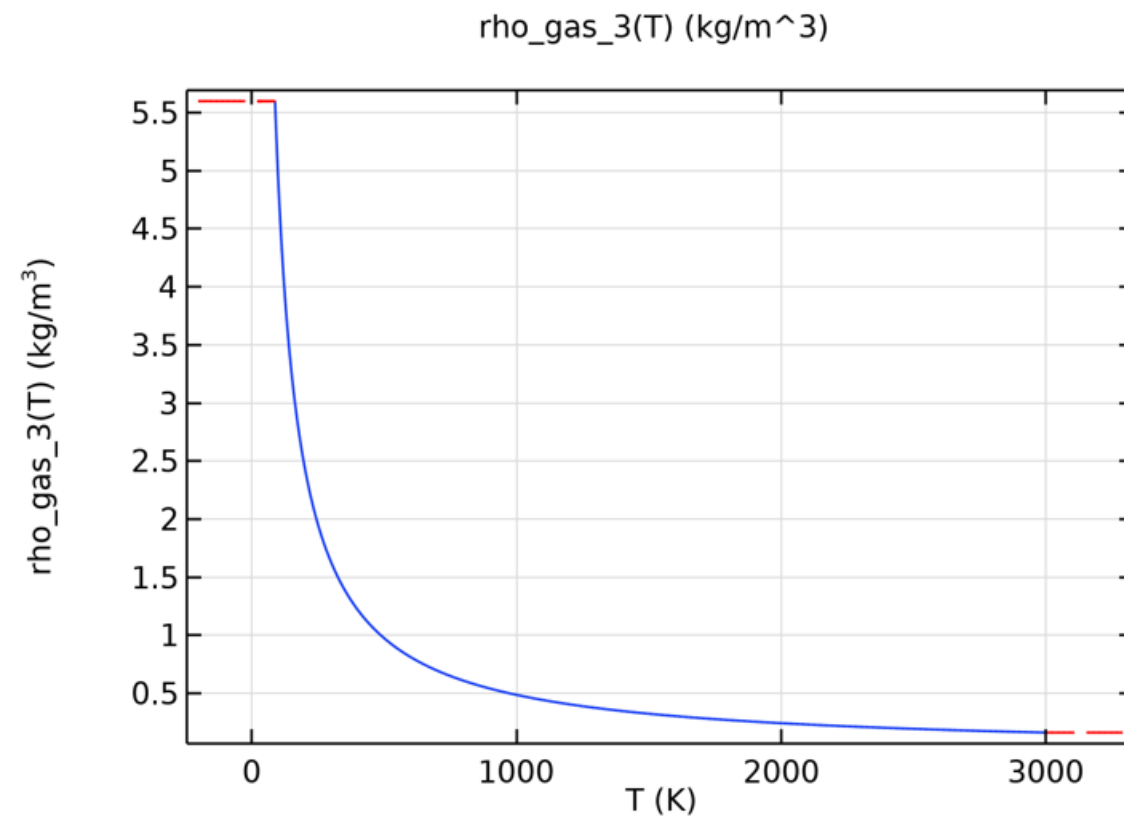

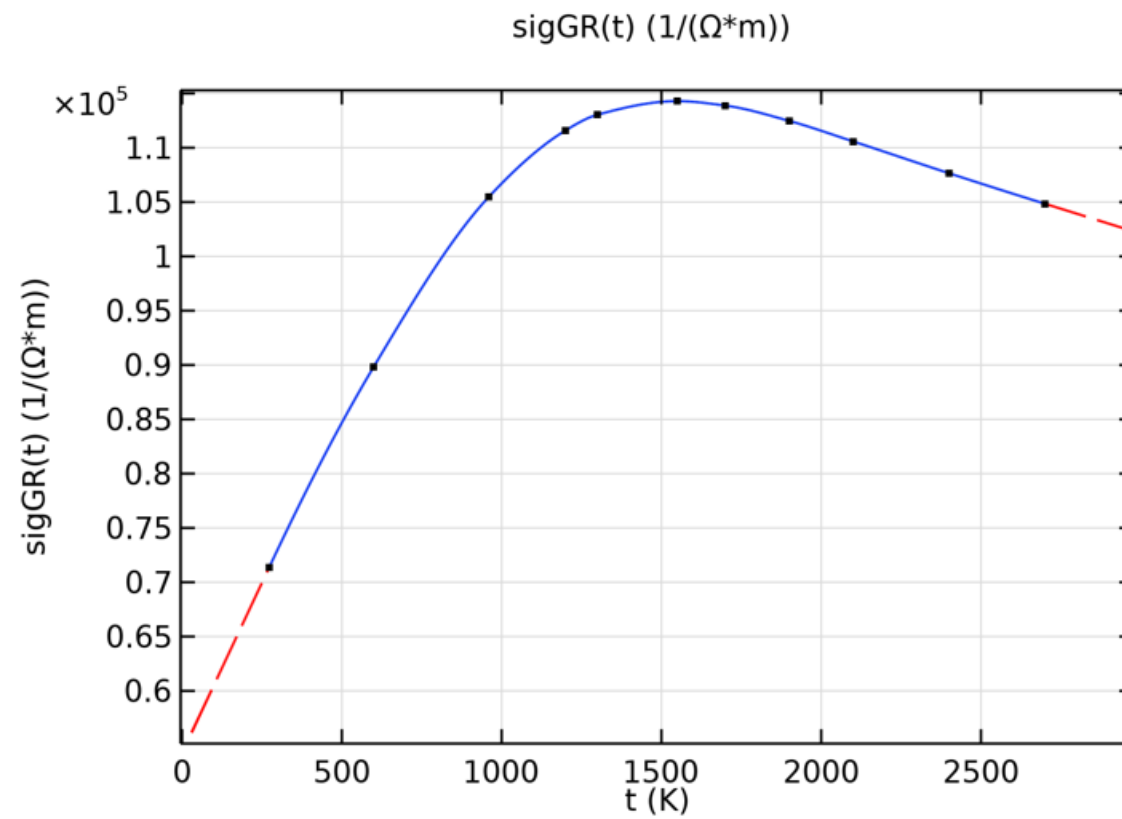

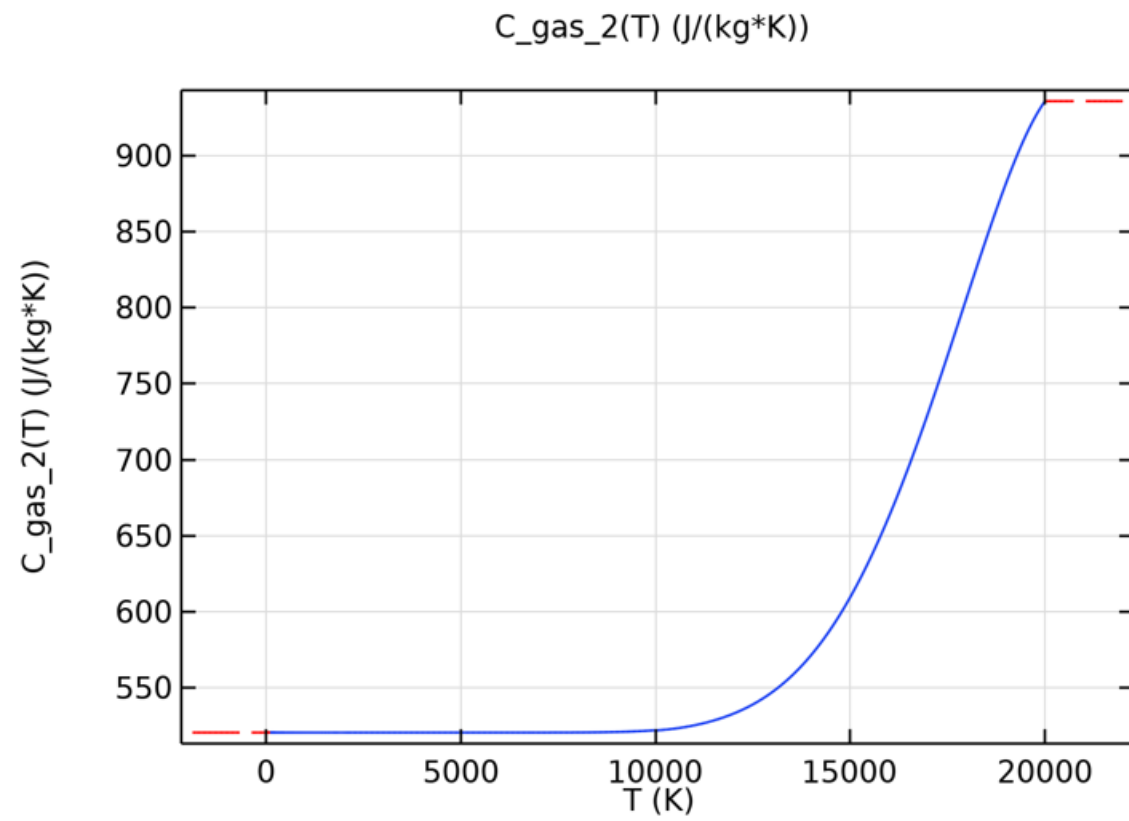

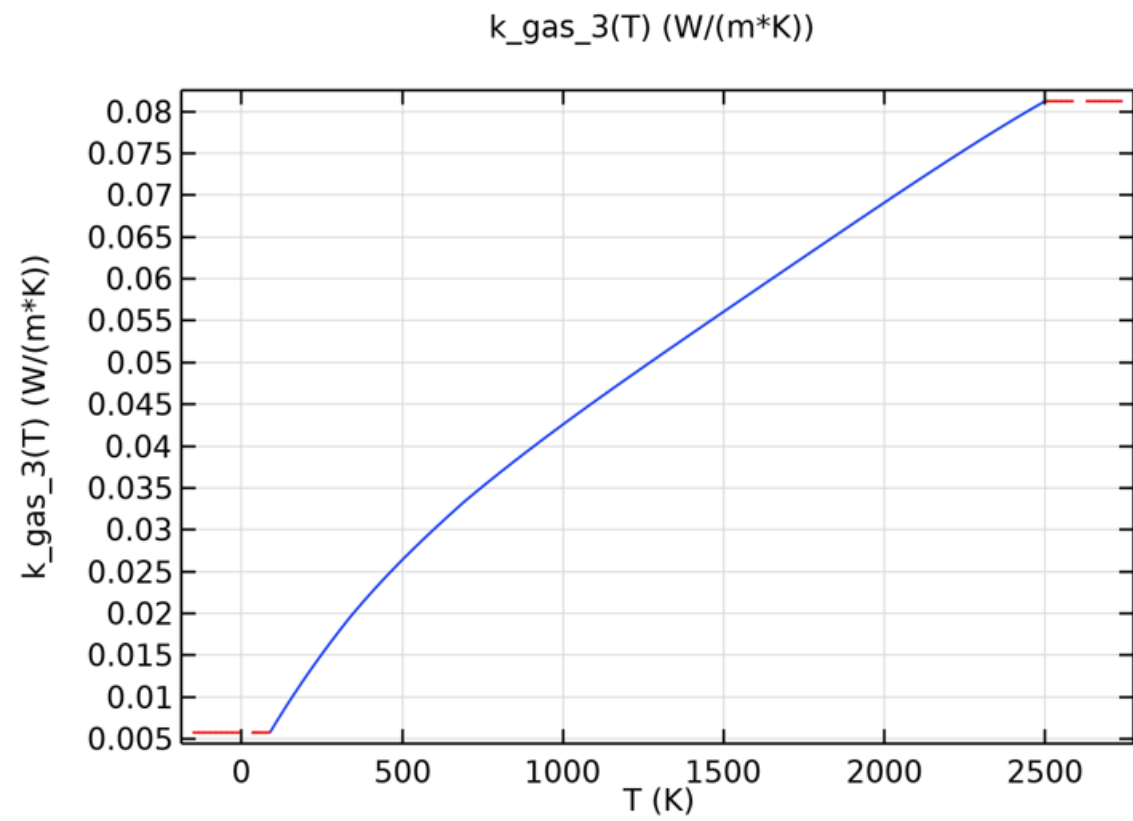

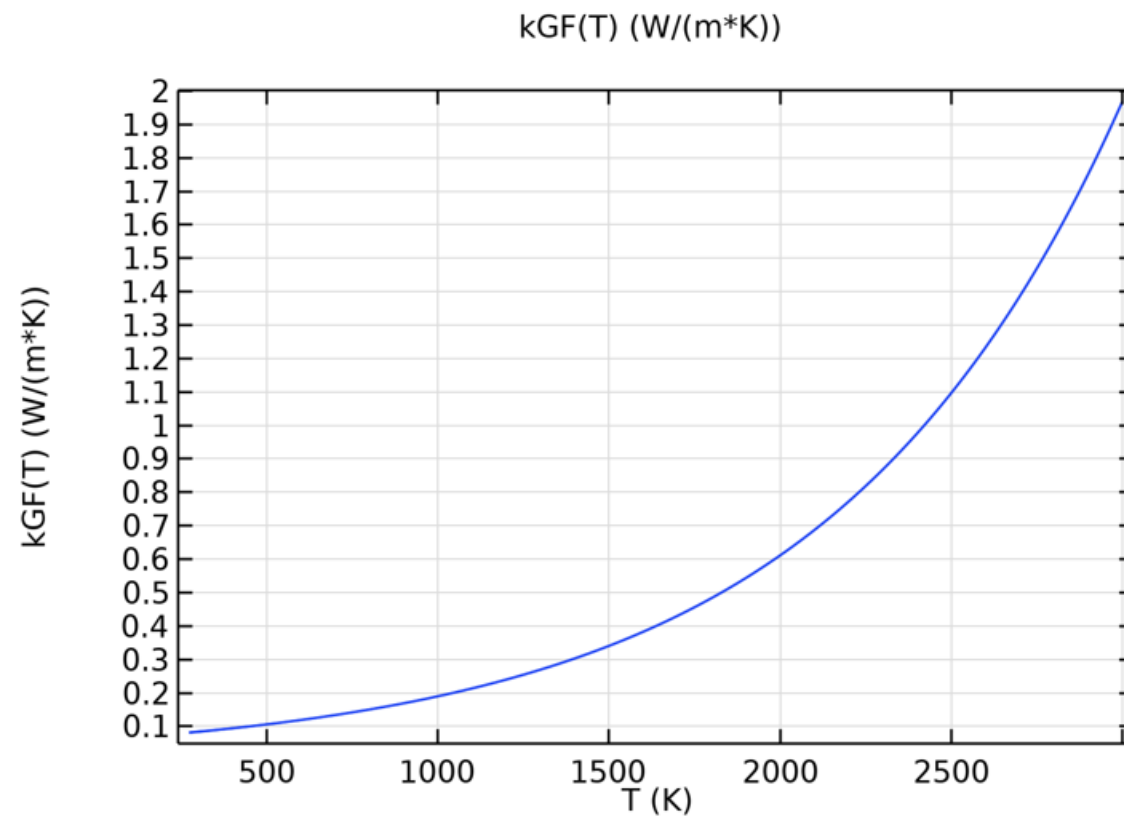

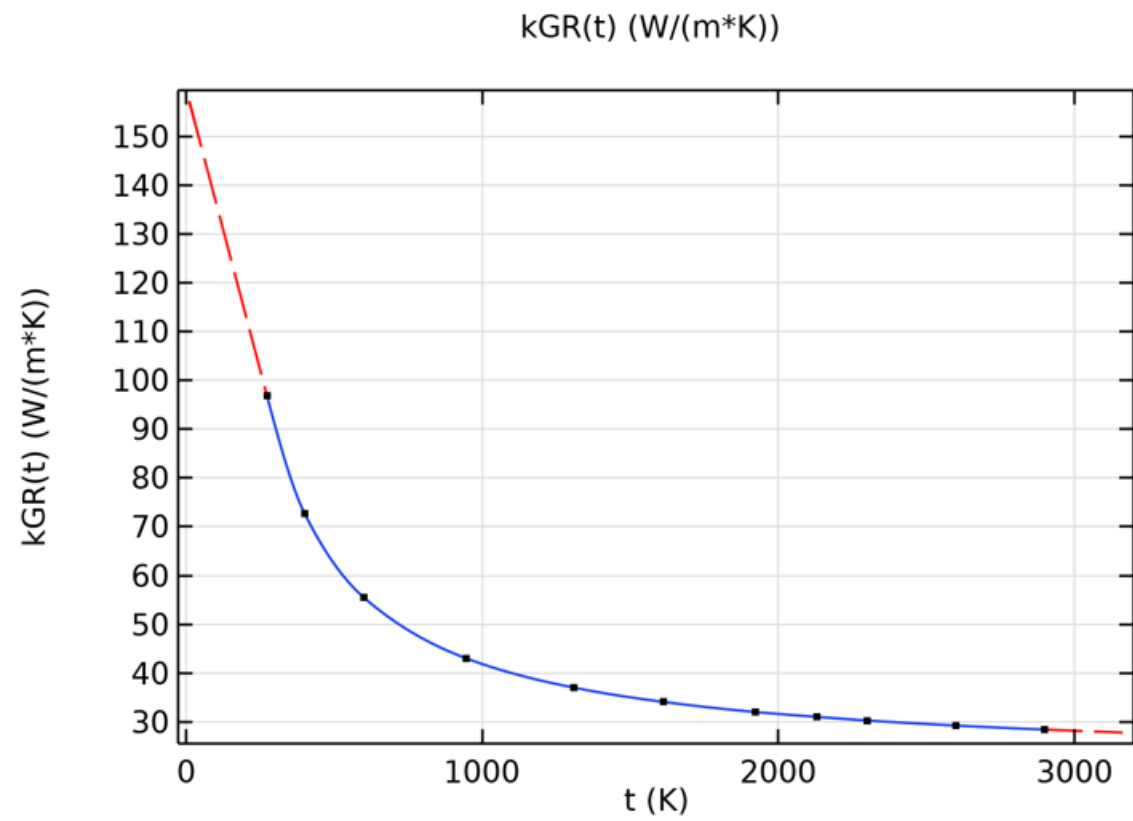

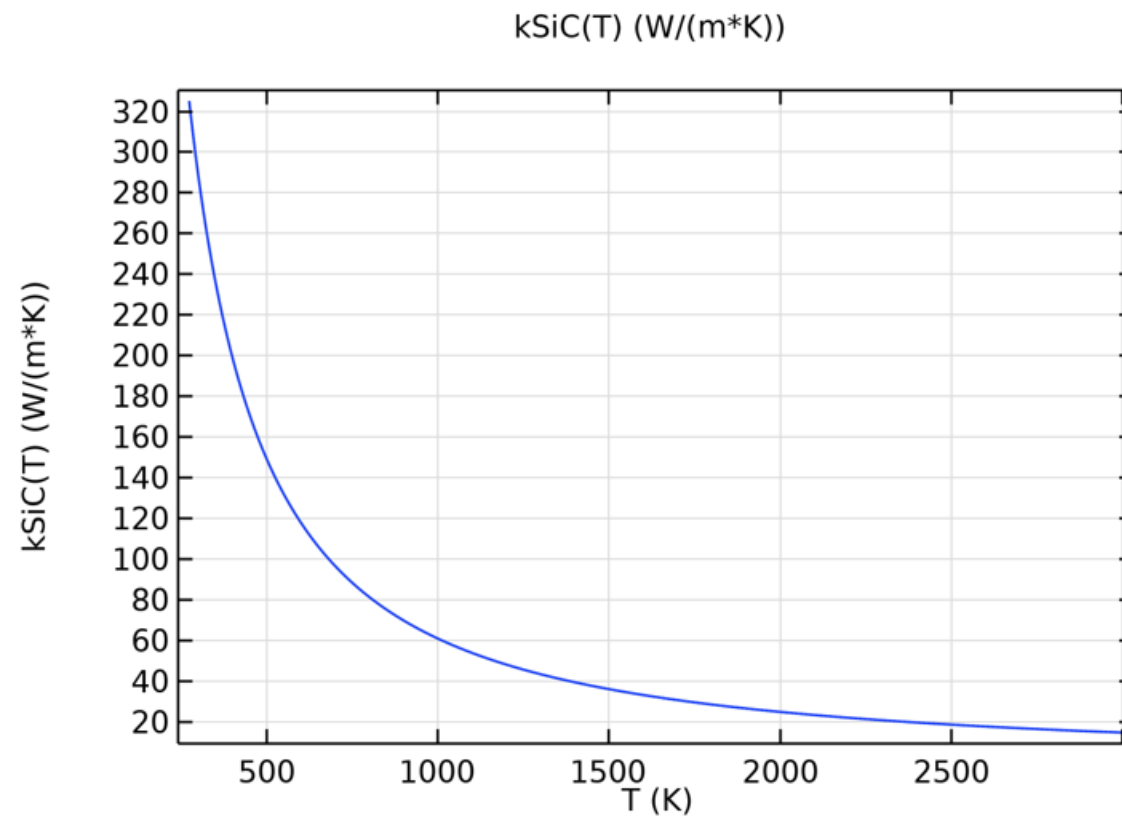

Supplement: Supplementary file 1 [file materials-16-00767-s001.zip › materials-2085973-supplementary.pdf]
